# On Vaccination

**Published:** 1804-06-01

**Authors:** John Ring

**Affiliations:** New Street, Hanover Square


### *To the Editors of the Medical and Phyſical Journal.*

GENTLEMEN,

IN the Remarks on the pustulous eruptions of animals, which you did me the favour to insert in the last Number of your Journal, there is a considerable error; the second paragraph, relative to the disease of turkies on the coast of Barbary, being put before the first. I therefore request, that the passage may be reprinted as follows:

" I am informed by Major Magra, who resided six years

---

years at Tunis, in the character of His Majesty's Agent and Consul General, that the same disease prevails among turkies on the coast of Barbary. Their heads swell, and are affected with pustulous eruptions, and they become blind. The disorder is supposed to be infectious."

"Major Magra was told it was the small-pox, and that it always proves fatal. He landed twelve turkies from Sicily, and they all died. It is impossible to breed them at Tunis, although fowls are very plenty there."

*New Street, Hanover Square,* I am, &c.

